# Taste cells depend on axon proximity to generate presynaptic sites

**DOI:** 10.1371/journal.pone.0325312

**Published:** 2025-06-03

**Authors:** Shannon M. Landon, Emily Holder, Amber Ng, Ryan Wood, Eduardo Gutierrez Kuri, Saima Humayun, Laura Bordallo Pinto, Lindsey J. Macpherson

**Affiliations:** 1 Department of Neuroscience, Developmental and Regenerative Biology, The University of Texas at San Antonio, One UTSA Circle, San Antonio, Texas, United States of America; 2 Brain Health Consortium, The University of Texas at San Antonio, One UTSA Circle, San Antonio, Texas, United States of America; Wrocław University of Environmental and Life Sciences: Uniwersytet Przyrodniczy we Wroclawiu, POLAND

## Abstract

The turnover and re-establishment of peripheral taste synapses is vital to maintain connectivity between primary taste receptor cells and the gustatory neurons which relay taste information from the tongue to the brain. Despite the importance of neuron-taste cell reconnection, the mechanisms governing synapse assembly in the taste bud are largely unknown. To determine whether nerve fiber connectivity is an initiating factor for the recruitment of presynaptic machinery in taste receptor cells, we use the expression of CALHM1 and Bassoon to identify presynaptic sites in type II (sweet, umami, bitter) and type III (sour) taste receptor cells, respectively. Under homeostatic conditions, the vast majority (>90%) of presynaptic sites are directly adjacent to nerve fibers (contacted). In the days immediately following gustatory nerve transection and denervation of taste buds, Bassoon and CALHM1 puncta are markedly reduced. This suggests that nerve fiber innervation is crucial for the recruitment and maintenance of presynaptic sites. During nerve fiber regeneration into the taste bud, presynaptic sites begin to replenish but are not as frequently contacted by nerve fibers as intact controls (35–54% compared to >90%). This reveals that taste cells rely on gustatory fiber innervation to organize presynaptic sites. Additionally, our finding that presynaptic sites are not as frequently contacted by regenerating axons suggests a model whereby trophic factors secreted by gustatory nerve fibers prompt taste receptor cells to produce and/or aggregate presynaptic specializations at the cell membrane prior to contact. This, in turn, may guide neurons to form mature synapses. These findings provide new insights into the mechanisms driving synaptogenesis and synaptic plasticity within the rapidly changing taste bud environment.

## Introduction

The mammalian taste bud contains a collection of mature and developing taste receptor cell (TRC) populations, as well as the peripheral axons of gustatory neurons. Together, they detect and then relay taste signals from the tongue to the brain. TRCs detect one of the 5 major taste modalities: sweet, bitter, salty, sour, and umami. TRCs that respond to sweet, bitter, and umami are classified as type II cells and have unique presynaptic structures whereby the neurotransmitter, ATP, is released through CALHM1/3 channels [1–4]. These type II TRC channels have been observed directly adjacent to afferent nerve fibers, facilitating precise, non-vesicular neurotransmission [2]. Type III cells, which detect sour and ionic stimuli, release serotonin through classical vesicular exocytosis [5–7]. While numerous synaptic markers have been evaluated for type III cells, Bassoon, a scaffolding protein in presynaptic active zones, has proven to be the only consistently reliable marker for identifying type III cell presynaptic sites [5,8–13].

Taste information is primarily relayed from TRCs via ATP signaling, activating the purinergic P2X2/P2X3 receptors specific to gustatory nerve fibers innervating the taste bud [14–18]. There is evidence showing serotonin plays a small role in taste signaling [19], however, the activation of P2X2/P2X3 receptors is necessary for every taste modality, including sour [14,16,20]. To relay taste signals, the peripheral axons of gustatory neurons associate closely with TRCs, often making more than one synaptic contact [21]. The synaptic contacts made between TRCs and gustatory neurons do not last long, as TRCs have a finite lifespan of approximately 10 days [22]. Older TRCs die off and are replaced by newly born TRCs, a frequent event commonly referred to as TRC turnover. Gustatory axons must re-connect with TRCs on a regular basis. Therefore, the formation and removal of synapses in the taste bud is quite common. This continual synapse turnover is critical for the integrity of taste sensation and taste related behaviors. Despite its importance, however, the mechanisms governing synapse formation in the taste bud are entirely unstudied.

Pre- and post-synaptic specializations must be aligned together accurately to create a synapse. While many studies have demonstrated that synaptic adhesion molecules (SAMs) are key components of synapse nucleation, forces that drive the assembly of SAMs at synapses and the guidance of the pre- and post-synaptic terminals to each other is not well understood [23,24]. It is likely that processes in both taste cells and gustatory neurons coordinate to organize synapse formation in the taste bud. Here, we probe the connectivity between gustatory neurons and TRC presynaptic proteins to interrogate the role of gustatory nerve fiber innervation and contact in synaptogenesis within the taste bud. Our goal is to determine whether TRCs rely on gustatory neuron innervation for the formation of presynaptic sites and whether neuronal contact triggers the assembly of presynaptic sites on TRCs. To accomplish this, we employed the use of presynaptic markers, Bassoon [8] and CALHM1 [1–4], and gustatory neuron marker, P2X2 [17,25], to observe gustatory nerve fiber innervation and contact frequencies with presynaptic sites on TRCs in normal and denervated conditions. We found that presynaptic sites are highly colocalized with gustatory nerve fibers, which then effectively vanish following denervation. When gustatory nerve fibers re-innervate the taste bud, presynaptic sites reappear on TRCs, though they are contacted far less frequently than intact controls. Thus, we demonstrate that TRCs depend on the close proximity of gustatory neurons to produce presynaptic sites, independent of direct contact. This finding provides a basis for mechanisms driving the continual re-establishment of synapses within the taste bud and grants novel insights into synaptogenesis in a highly plastic system.

## Results

### Most presynaptic specializations are “contacted” by nerve fibers

**Fig 1:**
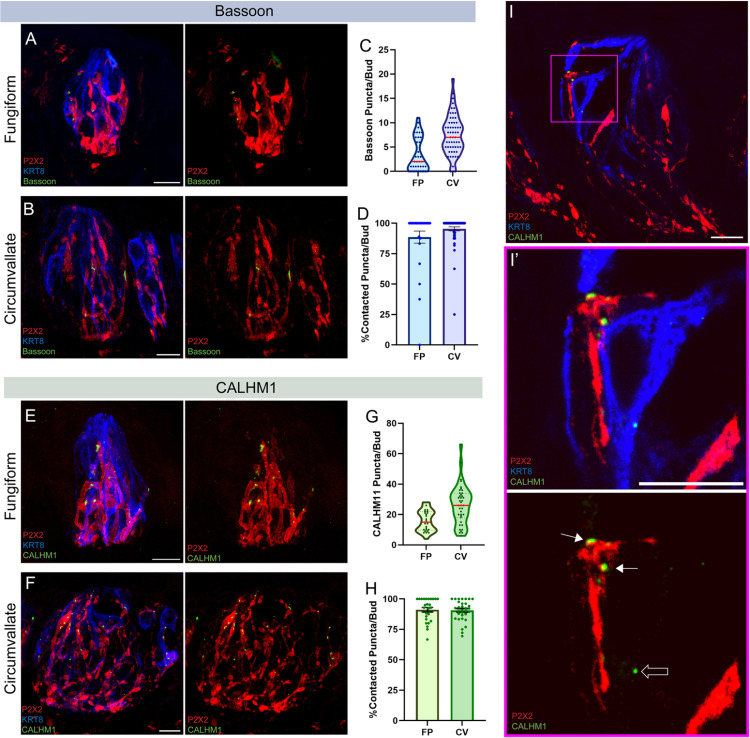
Bassoon and CALHM1 are frequently contacted by gustatory neurons. Immuno-labeling of P2X2 (red), KRT8 [mature TRCs (blue)], and Bassoon (green) in **A**) fungiform and **B**) circumvallate taste bud maximum intensity projections. **C**) Number of Bassoon puncta per taste bud in fungiform and circumvallate taste buds (red line: median). **D**) Percentage of Bassoon puncta that were contacted by P2X2-positive nerve fibers per taste bud. **C&D**) FP: N = 36 buds/4 mice, CV: N = 57 buds/4 mice. Immuno-labeling of P2X2 (red), KRT8 (blue), and CALHM1 (green) in **E**) fungiform and **F**) circumvallate taste buds. **G**) Number of CALHM1 puncta per taste bud in fungiform and circumvallate taste buds (red line: median). **H**) Percentage of CALHM1 puncta that were contacted by P2X2-positive nerve fibers per taste bud. **G&H**) FP: N = 26 buds/4 mice, CV: N = 39 buds/4 mice. **I**) Maximum intensity projection of a small subset (4 z-slices) of an immuno-labeled circumvallate taste bud using P2X2 (red), KRT8 (blue), and CALHM1 (green) staining. **I’**) Blown-up inset of the magenta box in (**I**) to highlight contacted (solid arrow) vs. uncontacted (hollow arrow) CALHM1 puncta. Scale: 10µm. Error bars: SEM.

Are TRC presynaptic sites always contacted by gustatory nerve fibers? Answering this question will establish the extent to which TRCs rely on gustatory neuron contact to form and/or maintain presynaptic sites (Fig 1). To determine this, we used immuno-staining of presynaptic sites on type II (CALHM1) and type III cells (Bassoon), observed as fluorescent puncta within the taste bud (Fig 1, S1 Fig). To quantify how many presynaptic sites were contacted by gustatory neurons under homeostatic conditions, gustatory axons were immunostained using the purinergic receptor P2X2. CALHM1 and Bassoon puncta that were overlapping with or directly touching P2X2 staining were categorized as “contacted puncta” and all others were characterized as “uncontacted”. Immuno-staining for cytokeratin 8 (KRT8), which is a marker for mature TRCs [26,27], was used to identify the taste bud area. Taste buds from both the anterior (fungiform papillae [FP]) and posterior (circumvallate papillae [CV]) tongue were analyzed.

On average, fungiform taste buds contained 3.4 (±0.58 SEM) Bassoon puncta per (18 µm) section and circumvallate tasted buds averaged 7.5 (±0.54 SEM) Bassoon puncta per section (Fig 1a–1c). CALHM1 puncta were more plentiful than Bassoon, with fungiform taste buds averaging 15.9 (±1.37 SEM) puncta per section and circumvallate taste buds containing an average of 26.5 (±2.06 SEM) puncta per section (Fig 1e–1g). The prevalence of CALHM1 puncta over Bassoon is expected due to the greater abundance of type II cells relative to type III cells in each taste bud [13,21,28].

We found that Bassoon puncta are contacted by nerve fibers at a frequency of 89% for fungiform and 95% for circumvallate taste buds (Fig 1d). Similarly, CALHM1 puncta are contacted by nerve fibers 91% of the time for both fungiform and circumvallate taste buds (Fig 1h). A small percentage (5–11%) of Bassoon and CALHM1 puncta are not contacted. This indicates that while nerve fiber contact is not absolutely necessary for TRCs to produce Bassoon and CALHM1, nerve contact may contribute to the formation and/or maturation of presynaptic sites in TRCs.

### Gustatory nerve transection leads to denervation of taste buds within 4 days

To determine whether TRCs depend on innervation to produce presynaptic sites, we first removed gustatory nerve fibers by nerve transection. Although the removal of peripheral gustatory neuron axons from taste buds is essential for this study, this has deleterious effects on the taste bud. Since gustatory nerve derived trophic factors, such as R-Spondin, are necessary to stimulate taste stem cells to replenish dying TRCs, nerve transection pauses TRC regeneration [29]. As a result, transection of the chorda tympani (CT) nerve results in a substantial decrease in taste bud number, taste bud size, and taste cell numbers in fungiform papillae [30–32]. Similarly, transection of the glossopharyngeal nerve results in almost complete loss of all circumvallate taste buds 42 days following transection [33,34].

**Fig 2:**
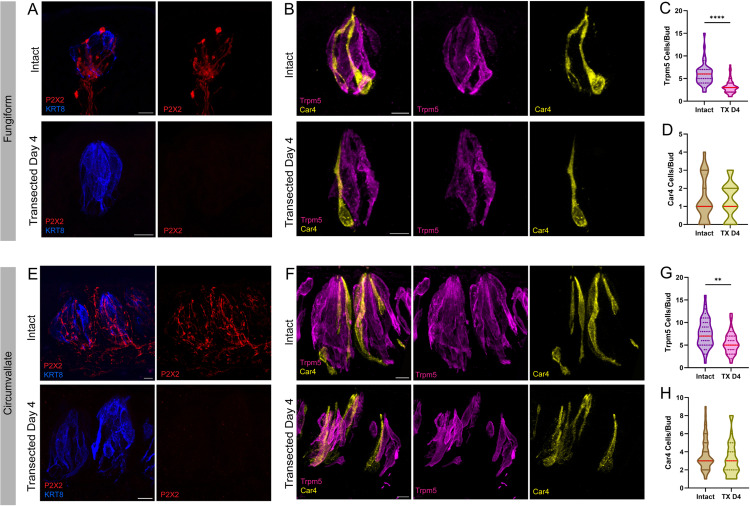
Within 4 days of nerve transection, taste buds are denervated. Comparison of immuno-labeled taste buds from intact control mice and mice four days post nerve transection (TX D4). **A&E**) Nerve fiber degeneration occurs within four days of nerve transection in (**A**) fungiform and (**E**) circumvallate taste buds, as determined by undetectable levels of P2X2 in transected mice. This loss of innervation is accompanied by a significant loss of type II cells, marked by Trpm5 (magenta), but not type III cells, marked by Car4 (yellow) in (**B**) fungiform and (**F**) circumvallate taste buds. Quantification of taste cell types in intact and transected mice reveals a significant loss of type II cells (**C&G**) while the loss of type III cells is not significantly different (**D&H**) in the transected groups (red line: median). Intact FP: N = 40 buds/4 mice, Transected FP: N = 50 buds/5 mice, Intact CV: 45 buds/3 mice, Transected CV: 35 buds/3 mice. Scale: 10µm. Significance calculated using Mann-Whitney U: **P < 0.0021, ****P < 0.0001.

Therefore, in order to analyze denervated taste buds with a minimal loss of TRCs, we needed to establish the earliest timepoint following nerve transection where afferent fibers have degenerated completely. To do this, we performed bilateral transection of either the CT or glossopharyngeal nerves to denervate the fungiform or circumvallate taste papillae, respectively. By the fourth day following surgery, gustatory nerve fibers (labeled by P2X2) were no longer observed in fungiform and circumvallate taste buds (Fig 2a,2e). To assess the extent of TRC loss 4 days post nerve transection, we used Trpm5 and Car4 antibodies to identify type II and type III cells, respectively. A significant loss of type II (Trpm5) cells in both fungiform (~47%) and circumvallate (~28%) taste buds was observed (Fig 2c,2g). Changes in the numbers of type III (Car4) cells were not significant in either fungiform (~3%) or circumvallate (~16%) taste buds at this time point (Fig 2d,2h). These findings are, for the most part, consistent with previous estimates of the lifespans of TRCs. Using pulse-chase EdU staining in circumvallate papillae, it was estimated that the half-life of type II TRCS is 8 days while the half-life of type III TRCs is 22 days [35]. Assuming exponential decay from these estimates, after 4 days, 29% of type II TRCs and 12% of type III TRCs would be expected to die from the natural rate of turnover in CV taste buds, without the addition of new TRCs. It is unclear whether the higher rate of loss of type II TRCs in the fungiform papillae we observed arises from an acceleration of cell death due to the loss of innervation or whether fungiform TRCs have a shorter lifespan than that of CVs. Future studies may clarify this point.

### Gustatory nerve denervation results in a significant reduction of presynaptic sites

**Fig 3:**
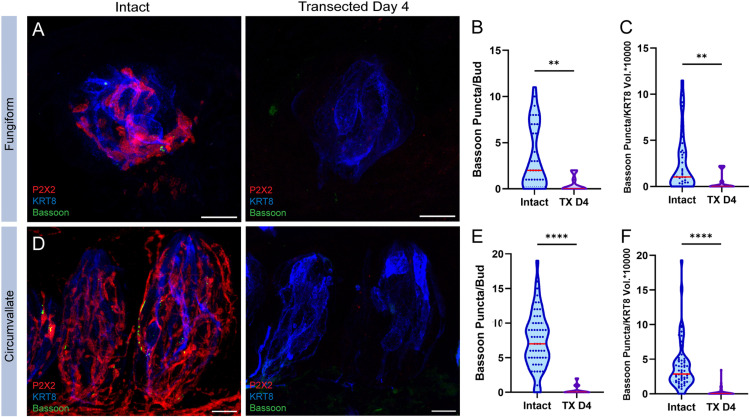
Bassoon puncta loss following nerve transection. Immuno-labeling of P2X2 (red), KRT8 (blue), and Bassoon (green) in intact and transected day 4 (TX D4), (**A**) fungiform and (**D**) circumvallate taste buds. Number of Bassoon puncta per taste bud in intact and transected (**B**) fungiform and (**E**) circumvallate taste buds (red line: median). The number of Bassoon puncta normalized to KRT8 volume per taste bud in intact and transected (**C**) fungiform and (**F**) circumvallate taste buds (red line: median). Data points in violin plots for intact controls (**B,E**) were reproduced from Fig 1c,1g. CV: intact N = 57 buds/4 mice, TX D4 N = 61 buds/4 mice. FP: Intact N = 36 buds/4 mice, TX D4 N = 11 buds/2 mice. Scale: 10µm. Significance calculated using Mann-Whitney U: **P < 0.0021, ****P < 0.0001.

Because of the high rates of contact between TRC presynaptic sites and gustatory nerve fibers (~90%), we hypothesized that innervation promotes or maintains presynaptic sites within the taste bud. Therefore, we predicted that loss of gustatory innervation within the taste bud would reduce the number and/or intensity of Bassoon and CALHM1 immunolabeled puncta. To test this, we performed bilateral CT nerve transections to eliminate fungiform innervation, and bilateral glossopharyngeal nerve transections to remove innervation in circumvallate taste buds. Strikingly, Bassoon immunolabeled puncta were almost undetectable in fungiform (0.45 ± 0.25 SEM) and circumvallate (0.31 ± 0.08 SEM) taste buds 4 days following nerve transection compared to intact controls (Fig 3a–3b, 3d–3e).

To account for taste cell loss following nerve transection, Bassoon puncta were normalized to the corresponding volume of mature TRCs per bud using KRT8 staining. This shows that the loss of Bassoon puncta is not a result of the reduction of mature taste cells due to normal TRC die-off in the absence of replenishment (Fig 3c,3f). Additionally, type III cell loss, specifically, cannot account for the ablation of Bassoon puncta, as there are still 85–95% of type III cells remaining 4 days following nerve transection (Fig 2d,2h).

**Fig 4:**
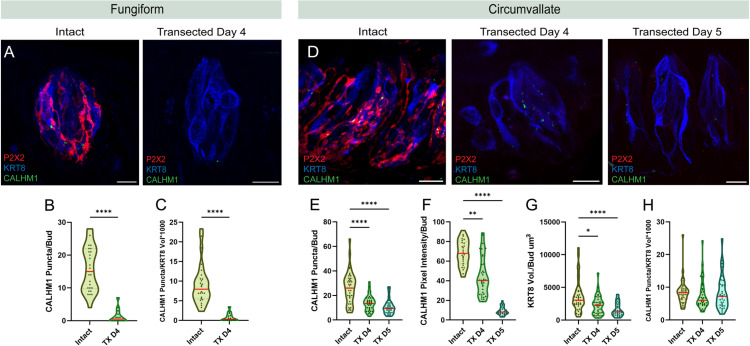
CALHM1 puncta loss following nerve transection. **A**) Immuno-labeling of P2X2 (red), KRT8 (blue), and CALHM1 (green) in intact and transected day 4 fungiform taste buds. **B**) Number of CALHM1 puncta per taste bud in intact and transected day 4 fungiform taste buds. **C**) Number of CALHM1 puncta normalized to KRT8 volume per taste bud in intact and transected fungiform taste buds. **(D)** Immuno-labeling of P2X2 (red), KRT8 (blue), and CALHM1 (green) in intact, transected day 4, and transected day 5 circumvallate taste buds. **E**) Number of CALHM1 puncta per taste bud in intact, transected day 4, and transected day 5 circumvallate taste buds. **F**) CALHM1 pixel intensity per taste bud in intact, transected day 4, and transected day 5 circumvallate taste buds. **G**) KRT8 volume per taste bud in intact, transected day 4, and transected day 5 circumvallate taste buds. **H**) Number of CALHM1 puncta normalized to KRT8 volume per taste bud in intact, transected day 4, and transected day 5 circumvallate taste buds. Data points in violin plots for intact controls (B,E) were reproduced from Fig 1c,1g. Median values in the violin plots are shown with a red line. FP: Intact N = 26 buds/4 mice, TX D4 N = 14 buds/2 mice. CV: Intact N = 39 buds/4 mice, TX D4 N = 36 buds/3 mice, TX D5 N = 34 buds/3 mice. Scale: 10µm. Significance calculated using Mann-Whitney U (B&C) and Kruskal-Wallis (F&G, H&I): *P < 0.0332, ****P < 0.0001.

Similarly, type II presynaptic sites are almost entirely lost following nerve transection. In fungiform papilla, CALHM1 puncta are strikingly reduced 4 days post nerve transection (1.36 ± 0.55 SEM) (Fig 4a,4b). This reduction is independent of mature TRC loss (Fig 4c) and cannot be attributed specifically to the loss of type II cells, given that they are reduced only by ~45% at that time point (Fig 2c). Circumvallate papillae, on the other hand, show a different pattern of CALHM1 puncta loss. CALHM1 loss was significant 4 days following transection compared to intact controls, however, a fair number of puncta persisted (13.5 ± 1.05 SEM, N:36 buds) (Fig 4d,4e). We extended our examination to 5 days following nerve transection and found that while more puncta are lost, some persist (10.5 ± 1.03 SEM, N:34 buds) (Fig 3d,3e). CALHM1 puncta loss mirrored the loss of mature taste cells (Fig 4g,4h) and type II cells (Fig 2g). Additional analysis revealed that even though the reduction in the numbers of CALHM1 puncta follows TRC loss, the fluorescence intensity of remaining CALHM1 puncta is significantly reduced (Kruskal-Wallis test) (Fig 4f). Thus, CALHM1 is indeed impacted by neuronal denervation in both circumvallate and fungiform taste buds. Together, these findings reveal that normal accumulation of presynaptic machinery in TRCs is dependent on nerve fiber innervation.

### CALHM1 and Bassoon mRNA expressions are not significantly changed following gustatory denervation

**Fig 5:**
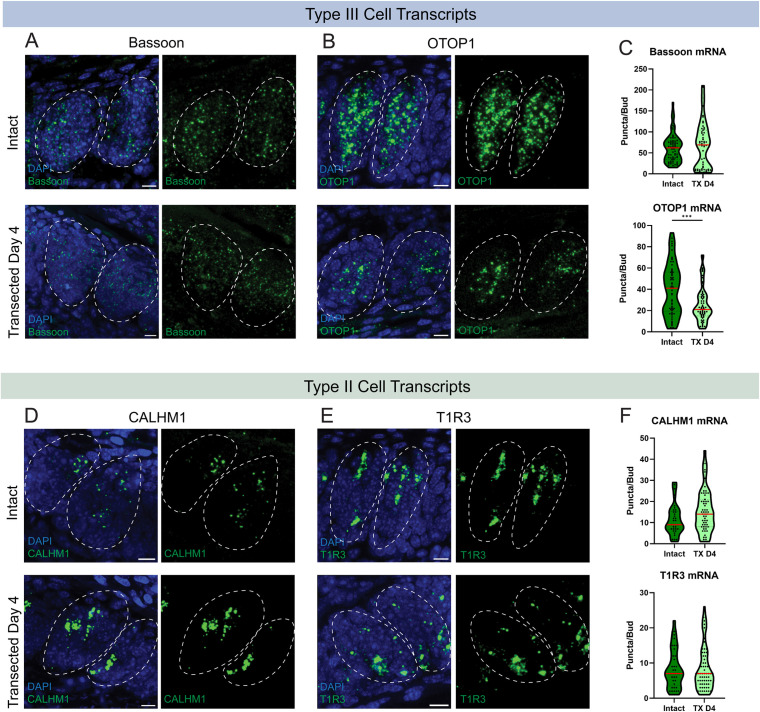
Transcription of taste receptors, OTOP1 and T1R3, and presynaptic proteins, CALHM1 and Bassoon, following gustatory denervation. Fluorescence in situ hybridization (FISH) labeling of **(A)** Bassoon and **(B)** OTOP1 in intact and transected day 4 circumvallate papillae showing Bassoon and OTOP1 mRNA transcripts as green puncta. **C**) Bassoon and OTOP1 transcripts quantified as puncta per bud in intact and transected circumvallate papillae (red line: median). FISH labeling of **(D)** CALHM1 and **(E)** T1R3 in intact and transected day 4 circumvallate papillae showing CALHM1 and T1R3 mRNA transcripts as green puncta. **F**) CALHM1 and T1R3 transcripts quantified as puncta per bud in intact and transected circumvallate papillae (red line: median). Dashed circles indicate the taste bud area. Bassoon Intact: N = 69 buds/3 mice. Bassoon TX: N = 55 buds/3 mice. OTOP1 Intact: N = 63 buds/3 mice. OTOP1 TX: N = 74 buds/3 mice. CALHM1 Intact: N = 34 buds/3 mice. CALHM1 TX: N = 60 buds/3 mice. T1R3 Intact: N = 40 buds/3 mice. T1R3 TX: N = 74 buds/3 mice. Scale: 10µm. Significance calculated using Mann-Whitney U: ***P < 0.0114.

The loss of CALHM1 and Bassoon immuno-staining in denervated taste buds could be the result of transcriptional down regulation and/or degradation or dispersal of the proteins within the taste cells. To test whether the transcription of Bassoon and CALHM1 is affected by gustatory innervation, we performed fluorescence in-situ hybridization (FISH) of Bassoon and CALHM1 along with taste receptor transcripts (T1R3 and Otop1) in intact and denervated CV taste buds (Fig 5). Four days following nerve transection, OTOP1 transcripts decline significantly while Bassoon transcripts remain largely unaffected (Fig 5a–5c). While there was not a large decline in type III cell numbers per bud 4 days following nerve transection (Fig 2h), it appears that the remaining cells downregulate transcripts for the sour sensing proton channel, OTOP1, and continue expressing transcripts for the presynaptic scaffolding protein, Bassoon. Type II cells, however, display a different pattern of transcription in response to denervation. We would expect a decline in transcripts for taste receptors in type II cells due to the 28% drop in cell number (Fig 2g). Contrarily, we found that T1R3 transcripts, which encode a portion of the heterodimeric receptor for sweet and umami type II TRCs, remain unchanged. The number of CALHM1 transcripts is not significantly different following denervation (Fig 5d–5f).

Despite the near-complete loss of immunohistochemical staining for CALHM1 and Bassoon proteins following nerve transection, denervated taste cells continue to produce transcripts for these presynaptic proteins. This indicates that the transcripts may not be undergoing translation. Another possibility is that the transcripts are translated and folded into proteins, but the proteins remain diffuse throughout the membrane, waiting for the right signals to activate aggregation at presynaptic sites. Either way, it appears that the loss of presynaptic protein aggregation at the membrane following nerve fiber denervation is not a result of transcriptional down regulation of presynaptic proteins. The mechanism by which nerve fibers enable taste cells to accumulate presynaptic sites is, therefore, post-transcriptional.

### Gustatory neuron re-innervation following chorda tympani nerve transection

Early studies documenting the regeneration, innervation, and functional recovery of the CT nerve following injury in gerbils found that there was a delay between taste bud innervation and functional recovery of CT nerves [36,37]. Taste responses began to return in CT recordings about 12 days following nerve crush [37], whereas nerve re-innervation was observed as early as 9- and 10-days following nerve crush [36]. This delay could indicate that synaptic contact is not established immediately following nerve re-innervation into the taste bud. In mice, it has been shown that taste responses begin to reappear about 3 weeks following CT nerve crush. However, to our knowledge, there are no existing reports mapping the early timing of CT nerve re-innervation after injury. Therefore, we used in-vivo and ex-vivo imaging techniques to observe nerve re-innervation following CT nerve transection.

**Fig 6:**
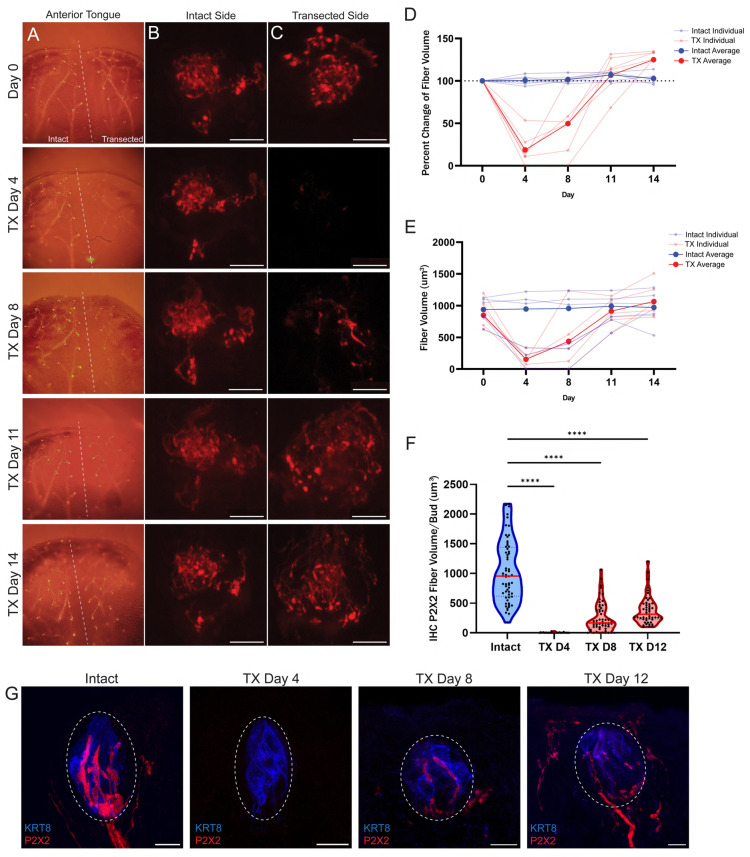
Regrowth of the chorda tympani nerve into fungiform papilla following nerve transection. Gustatory nerve regrowth was tracked in fungiform papilla, in-vivo and ex-vivo, following CT nerve transection. **A-E**) Phox2b-cre;Ai9 animals that underwent unilateral CT nerve transection were used for intravital imaging of the anterior tongue and fungiform papillae to visualize gustatory nerve innervation. **A**) Epifluorescent images of the anterior portion of the tongue over the course of 14 days, with a dashed line annotating the midline separating the intact left side and the CT transected right side. 2-photon images of the same taste bud were imaged over 14 days on the (**B**) intact side and (**C**) transected side of the tongue. **D&E**) Nerve fiber volume was quantified in intact (blue) and transected (red) taste buds over time and is shown as (**D**) percent of starting volume from day-0 and (**E**) absolute nerve fiber volume (N = 12 buds from 2 mice, 6 from intact and 6 from transected). **F**) Gustatory marker, P2X2, was quantified within the taste bud via Immunohistochemistry (IHC) in intact and bilateral CT nerve transected mice 4-, 8-, and 12-days following surgery (N = 50-60 buds/condition). Median values in the violin plots are shown with a red line. **G**) Gustatory neuron (P2X2) volume from intact and transected taste buds was quantified only from the taste bud area positive for KRT8 staining, indicated with white dashed circles. Scale: 10 µm. Significance calculated using Kruskal-Wallis: ****P < 0.0001.

To observe regenerating gustatory neurons, we monitored nerve regrowth in individual fungiform taste buds using intravital 2-photon imaging of Phox2b-Cre;Ai9 mice that had undergone unilateral CT nerve transection. In these animals, gustatory neurons and their axons are marked with bright red fluorescence. Images of the same taste buds were captured before and 14 days following unilateral nerve transection. Following CT nerve cut, nerve fibers had largely dissipated by day 4 and began to regenerate and re-innervate taste buds by day 8 (Fig 6a–6e). Interestingly, we found that nerve fiber volume began to return to normal levels by day 11 and showed even higher volumes by day 14. Upon further analysis, it appears that the rapid return of gustatory neurons may be located outside of the taste bud proper, in the perigemmal space. Because it was difficult to distinguish the taste bud area from the surrounding epithelium, the sampling area for volume analysis included the entire fungiform papillae rather than just the taste bud area. On days 11 and 14, the nerve fibers appear to be more spread out compared to the intact side (Fig 6b,6c), which may be due to more fibers innervating the perigemmal space rather than within the actual taste bud.

To confirm the 2-photon analysis, fungiform taste buds from intact and transected day 4, 8, and 12 were immunostained and imaged, revealing that gustatory neuron re-innervation does indeed begin to occur approximately 8 days following nerve transection (Fig 6f). Additionally, we demonstrated that P2X2 is a suitable antibody marker for regenerating axons by co-labeling gustatory neurons with genetically driven expression of td-Tomato in gustatory neurons using Phox2b-Ai9 mice (S2 Fig). Regenerating nerve fiber volume within taste buds of day 12 transected mice (determined by KRT8 staining) is significantly less than intact controls (Fig 6f). In the immunostained images, we confirm that there are indeed numerous branches of re-innervating gustatory neurons in the perigemmal space (Fig 6g). Therefore, the total volume of gustatory neurons within fungiform papillae returns by day 12 (Fig 6a–6e), but the portion that innervates only the taste bud area remains less than intact controls by day 12 (Fig 6f,6g). The data shown here are consistent with previous reports that regenerating gustatory neuron volume within the taste bud is less than intact taste buds even 8 weeks or longer following CT nerve injury [38,39].

### Chorda Tympani nerve re-innervation stimulates the return of Bassoon and CALHM1 independent of nerve fiber contact

**Fig 7:**
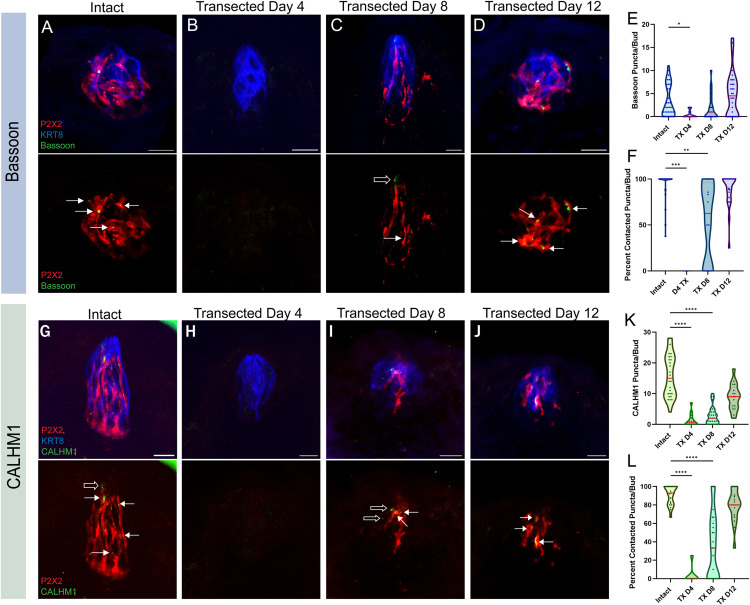
Bassoon and CALHM1 puncta are contacted less frequently by gustatory neurons during re-innervation of taste buds. Immuno-labeling of P2X2 (red), KRT8 (blue), and Bassoon (green) in (**A**) intact, (**B**) transected day 4, (**C**) transected day 8, and (**D**) transected day 12 fungiform taste buds. Solid arrows indicate contacted puncta while hollow arrows indicate uncontacted puncta. **E**) Number of Bassoon puncta per taste bud in each condition. **F**) Average percentage of Bassoon puncta that are contacted by P2X2-positive nerve fibers per taste bud in each condition. **E&F**) Intact: N = 36 buds/4 mice, TX D4: N = 11 buds/2 mice, TX D8: N = 25 buds/3 mice, TX D12: N = 32 buds/4 mice. Immuno-labeling of P2X2 (red), KRT8 (blue), and CALHM1 (green) in (**G**) intact, (**H**) transected day 4, (**I**) transected day 8, and (**J**) transected day 12 fungiform taste buds. Solid arrows indicate contacted puncta while hollow arrows indicate uncontacted puncta. **K**) Number of CALHM1 puncta per taste bud in each condition. **L**) Average percentage of CALHM1 puncta that are contacted by P2X2-positive nerve fibers per taste bud in each condition. **K&L**) Intact: N = 26 buds/4 mice, TX D4: N = 14 buds/2 mice, TX D8: N = 32 buds/4 mice, TX D12: N = 17 buds/4 mice. Scale: 10µm. Median values in the violin plots are shown with a red line. Significance calculated using Kruskal-Wallis: *P < 0.0332, **P < 0.0021, ***P < 0.0002, ****P < 0.0001.

To assess the effect of nerve re-innervation on Bassoon and CALHM1 accumulation in TRCs, we collected tissues from intact and CT transected day 4, 8, and 12 mice for immunostaining to determine when synapses are established (Fig 7). Consistent with our earlier findings, Bassoon and CALHM1 puncta were almost entirely abolished on the 4th day following nerve transection (Fig 7e,7k). On day 8, when nerve fibers begin to return to the taste bud, Bassoon and CALHM1 also began to come back (Fig 7e,7k). By day 12, median Bassoon puncta per bud exceeds that of intact controls, though the increase is not significant (Fig 7d,7e), while CALHM1 puncta had not yet returned to normal levels (Fig 7j,7k).

To determine whether nerve fiber contact is necessary for Bassoon and CALHM1 accumulation in taste cells, we quantified the percentage of Bassoon and CALHM1 that are contacted by P2X2 nerve fibers per taste bud during nerve re-innervation. When nerve fibers begin to re-innervate the taste bud 8 days post nerve transection, we found that Bassoon and CALHM1 puncta are contacted by P2X2 nerve fibers significantly less than intact controls (Fig 7f,7l). Nerve fiber contact does not return to near-normal levels until 12 days following nerve transection (Fig 7f,7l). These findings illustrate how the accumulation of Bassoon and CALHM1 proteins depends on the presence of nearby nerve fiber innervation, but not direct nerve contact. If nerve fiber contact was indeed necessary for taste cells to accumulate presynaptic sites, the rate of contacted Bassoon and CALHM1 puncta would remain at a high level, even at the early stages of re-innervation. Thus, the mechanisms driving synapse formation are likely initiated by signaling molecules secreted by the gustatory axon, rather than membrane-bound protein interactions, prompting the TRCs to form presynaptic sites.

## Discussion

These data reveal novel insights into the intricate mechanisms governing synaptogenesis and synaptic plasticity within the ever-evolving taste bud environment. By associating TRC presynaptic sites with gustatory nerve endings, we have uncovered the importance of gustatory nerves in driving synapse formation within the taste bud. Under normal conditions, presynaptic sites (marked by Bassoon and CALHM1) are present and frequently contacted by gustatory nerve fibers. The small population of uncontacted presynaptic sites may represent new or maturing TRCs that are preparing to synapse with a gustatory fiber or may be transiently remodeling their synaptic contacts with fibers. From recent publications [40–42], and our own observations of gustatory fibers using intravital imaging [43], we find that gustatory nerve axons are much more dynamic within the bud than was previously assumed. However, transection of the chorda tympani and glossopharyngeal nerves causes a significant decrease in Bassoon and CALHM1 puncta numbers and intensity, the extent of which cannot be explained solely by the loss of TRCs in denervated taste buds. Since mRNA levels for Bassoon and CALHM1 are not significantly changed by nerve transection, the loss of immunoreactivity suggests that these proteins disaggregate or degrade. A caveat of using Bassoon and CALHM1 immunoreactivity as a proxy for presynaptic sites is that we are likely underestimating the number of synapses in TRCs. Fluorescently detectable puncta may represent a subset of presynaptic sites that are more mature, and/or have recruited more Bassoon or CALHM1 protein to the active zone. Re-innervation experiments reveal that as the CT nerve regrows, Bassoon and CALHM1 puncta begin to reappear in the taste buds, though they are not contacted as frequently by the newly innervating nerve fibers. This suggests, either directly or indirectly, that the initial stages of nerve fiber regrowth can stimulate taste cells to produce presynaptic sites independently of direct nerve fiber contact. We hypothesize that there are neuronally derived trophic factors (perhaps R-Spondin [see below discussion]) that prompt taste cells to form presynaptic sites; however, further research is necessary to substantiate this theory.

Taste bud homeostasis depends on a complex interplay between taste receptor cells and gustatory neurons. In fact, when nerve fibers are transected, taste buds slowly deteriorate because taste cell regeneration is interrupted. TRCs can only replace themselves when there is a steady supply of neuronally derived R-Spondin [29]. The Wnt pathway in taste stem cells is positively regulated by R-Spondin through LGR5/6 receptors [29,44–46]. Thus, neuronal supply of R-Spondin stimulates the replacement of old taste cells. It is tempting to hypothesize that R-Spondin may also stimulate TRCs to produce presynaptic sites. If this hypothesis holds true, administering exogenous R-Spondin after nerve transection could potentially rescue the loss of presynaptic sites in taste cells. However, if R-Spondin is not the molecular trigger for presynaptic site production, this experiment could be repeated using any other putative neuronally derived trophic factor.

The capacity for gustatory nerve fibers to trigger TRC organization of presynaptic sites is only one piece of the puzzle. The precise mechanism by which TRCs and gustatory neurons coordinate and align their pre- and postsynaptic terminals to nucleate a synapse is another intriguing question. In general, synapse formation is thought to be orchestrated first by axon guidance mechanisms, positioning the axon adjacent to its target, then synaptic adhesion molecule (SAM) compatibility determines which neurons form synapses at what location [24]. These trans-synaptic adhesion molecules dictate the formation and activity-dependent function of synapses [47]. There are many known SAMs postulated to initiate synapse formation, though, none are documented in taste cells or gustatory neurons to interact in the trans-synaptic space. Gustatory neurons express GABA receptor a1 [48,49], a SAM that has been shown to interact with neurexins on the presynaptic cell within the hippocampus [50]. Additionally, sweet and bitter TRCs express semaphorin7a and -3a, respectively, to guide gustatory neurons to make appropriate and specific connections [51], while protocadherin-20 has been proposed to guide gustatory neurons to connect specifically to sweet and umami cells [52]. These molecular tags may help guide gustatory neurons to specific taste cells, though how gustatory nerve fibers coordinate with TRCs to form a synapse is yet unclear.

Release of neurotransmitter from TRCs may also play a role in guiding gustatory neurons to form synapses with specific partners. Because type II and type III cells release distinct neurotransmitters, ATP and serotonin respectively [6,14,17], it’s possible that ATP and serotonin release from TRCs informs gustatory neuron terminals to preferentially synapse with either type II or type III cells. Gustatory axon terminal arbors preferentially synapse with either type II (>95%) or type III (90%) cells, rarely both, as demonstrated in an analysis of 3D reconstructed circumvallate taste bud electron micrographs [21]. Therefore, it would be interesting to determine whether pharmacological blockade of purinergic and/or serotonergic receptors have any effect on the formation of specific synaptic connections.

The addition and removal of dendritic spines over time have been observed in the adult mouse brain [53]. Roughly 40% of all synapses turnover in a 5-day period within the hippocampus [53]. These studies were possible since gross axonal and dendritic structures are relatively stable [24]. In the taste bud, however, the peripheral endings of gustatory neurons are highly plastic, and synapses cannot be determined by terminal branch phenotypes. Anatomical analysis of gustatory nerve endings shows diverse morphologies including branch retraction and sprouting, indicating states of plasticity [54]. Moreover, intravital studies reveal that gustatory neurons exhibit remarkable plasticity [41,43], with peripheral arbors averaging a branch extension or retraction every 8 hours [41]. Because of this rapid plasticity, the ability to observe synapse turnover in the taste bud remains a challenge.

Indeed, tracking GFP-tagged Bassoon in vivo could be a useful tool to further define synapse formation in the taste bud. A study analyzing microtubule dependent assembly of presynaptic active zones used a GFP-tagged bassoon model to track the assembly of presynaptic sites [55]. Using this model to observe Bassoon trafficking within the taste bud in vivo could shed light on intracellular programs that drive presynaptic assembly.

Here, we have demonstrated basic principles of synapse formation in the mammalian taste bud, revealing that axon proximity influences the presence and maintenance of synapse-related proteins in TRCs. This fundamental mechanism provides a foundation for future studies investigating synapse formation within taste buds. Moreover, mechanisms of synapse formation and plasticity in the peripheral taste system can provide insights into other synapse-forming epithelial sensory cells, such as hair cells and Merkel cells, that have been proposed to descend from the same embryonic origin [56]. Extraoral chemosensory cells in the alimentary tract, known as enteroendocrine cells, share striking similarities with TRCs and have been shown to make direct synaptic contacts with vagal afferents [57]. Thus, understanding synapse formation in taste buds can greatly advance our knowledge of broader synaptic mechanisms in other epithelial sensory cells.

## Materials and methods

### Animals

All procedures were conducted in accordance with US National Institutes of Health (NIH) guidelines for the care and use of laboratory animals and were approved by the University of Texas at San Antonio IACUC, approval number MU111. Both male and female mice were used in this study. For immunohistochemical experiments of sectioned tongue tissues, C57/b6 mice (Jax Strain #000664) were used along with Phox2b-Cre;Ai9 double transgenic mice (Jax Strains #016223, #007909). All mice were between 3 and 8 months old; weighing between 18 and 35g. Food and water were available ad libitum. The mouse colony was maintained on a regular 12/12 h light-dark cycle. Animal numbers are reported in the figure legends.

### Nerve transection surgery

Aseptic bilateral nerve sectioning of the glossopharyngeal nerve or the chorda tympani (CT) nerve were performed in separate cohorts of mice. Mice were placed under aerosolized isoflurane anesthesia at 5% in oxygen (4L/min) for induction and 1–3% in oxygen (0.5L/min) for maintenance. Body temperature was maintained with a heating pad. The CT nerve was approached ventrally in the neck and transected after it bifurcates from the lingual nerve with severed ends left in place as previously described [58,59]. For glossopharyngeal nerve transection, the nerve was accessed ventrally through the neck and visualized by retracting the omohyoid and sternoid muscles medially and the digastric muscle and sublingual/submandibular salivary glands laterally. The glossopharyngeal nerve was located inferiorly to the hypoglossal nerve and lateral to the pharyngeal branch of the vagus nerve, where it was then transected and the ends left in place [33]. The wound was closed with discontinuous sutures. Meloxicam ER and buprenorphine ER analgesics were given prior to surgery and animal well-being was monitored in the days following surgery. Mice were then euthanized at various timepoints following surgery, as outlined in the text.

### Immunohistochemistry

Mice were euthanized through CO2 inhalation followed by trans-cardial perfusion with 20mL of 1x PBS followed by 10mL of 4% paraformaldehyde solution in 1x PBS. Because CALHM1 and Bassoon primary antibodies were raised in mouse, complete perfusion was critical to reduce background staining. Tongues were extracted and cryoprotected in 30% sucrose at 4 °C overnight. The tongues were then dissected to separate the circumvallate papillae (CV) and the anterior 2/3 of the tongue where the fungiform papillae (FP) are located. The anterior 2/3 of the tongue was then cut sagittally to split it into left and right halves. The left and right tongue tip and the CV were embedded in OCT compound and stored at −80 °C. Samples were cryo-sectioned at 18 μm thickness and mounted directly onto anti-frost slides. Sections of the CV and tongue tips underwent antigen retrieval by incubating in sodium citrate buffer (10mM Sodium Citrate, 0.05% Tween 20, pH:6) in an 80^o^C water bath for 20 minutes. The slides were removed from the sodium citrate buffer and allowed to cool to room temperature before washing with 1x PBS for 5 minutes. The slides were then dried along the edges before applying a hydrophobic barrier and dried for 5 more minutes. Blocking buffer was then applied to the sections: first, 5% donkey serum with 0.3% triton-x100 in 1x PBS at 25^o^C for 30 minutes, then blocked again with M.O.M.® (Mouse on Mouse) Blocking Reagent (MKB-2213–1) diluted to 1 drop in 1mL 1x PBS at 25^o^C for 30 minutes. Primary antibodies (Table 1) were diluted in 5% donkey serum with 0.3% triton-x100 in 1x PBS overnight at 4 °C. After 3 × 5-min washes with 1x PBS with 0.1% triton-x100, secondary antibodies (Table 1) were diluted to 1:1000 in 5% donkey serum with 0.3% triton-x100 in 1x PBS and incubated for 4 hr at 4^o^C. The slides were then washed for 3 × 5 min with 1x PBS with 0.1% triton-x100 and then mounted with VECTASHIELD® Antifade Mounting Medium (Vector Laboratories [H-1000–10]) and sealed with clear nail polish.

**Table 1:** Primary and secondary antibodies used for immunohistochemistry of taste tissue.

Primary Antibody/Host	Dilution	Company/Catalog #	RRID	Secondary Antibody
P2X2/Rabbit	1:500	Alomone Labs/ Cat #APR-003	AB_2040054	Alexa-fluor 594 AffiniPure Donkey Anti-Rabbit IgG (H + L)
Troma (KRT8)/Rat	1:1000	DSHB/ Cat # TROMA-I	AB_531826	Alexa-fluor Plus 405 Donkey anti-Rat IgG (H + L)
Bassoon/Mouse	1:500	Enzo/ Cat# ADI-VAM-PS003-F	AB_2313990	Alexa-fluor 488 AffiniPure Donkey Anti-Mouse IgG (H + L)
CALHM1/Mouse	1:50	Millipore Sigma/ Cat # MABN2521	AB_3096921	Alexa-fluor 488 AffiniPure Donkey Anti-Mouse IgG (H + L)
Trpm5/Rabbit	1:1000	Provided by Zuker Lab, Columbia University		DyLight 405 AffiniPure Donkey Anti-Rabbit IgG (H + L)
Car4/Goat	1:200	R&D Systems/ Cat # AF2414	AB_2070332	Alexa-fluor 647 AffiniPure Donkey Anti-Goat IgG (H + L)

### In situ hybridization

Mice were euthanized through CO2 inhalation and cervical dislocation. The CV of the tongue was quickly dissected, placed in ice-cold OCT medium, and flash frozen in dry ice. Fresh frozen tissues were stored in −80 °C until ready to process. Samples were cryo-sectioned at 15 μm thickness and mounted directly onto anti-frost slides. The protocol for Manual RNAscope® Assay for fresh/frozen tissues by ACD was followed using RNAscope® Multiplex Fluorescent V2 detection kit (Cat No. 323110). The probes used were RNAscope™ Probe- Mm-CALHM1 (Cat No. 558951-C1) and RNAscope™ Probe- Mm-Bsn-C1 (Cat No. 1119681-C1). Following probe and amplifier hybridization, DAPI was applied to the slides for 30 seconds. Stained slides were then mounted with coverslips using VECTASHIELD® Antifade Mounting Medium (H-1000–10) and sealed with clear nail polish.

### Confocal image acquisition and quantification

A Zeiss 710 confocal microscope was used with the 63x objective to image the tongue sections for individual taste buds. Whole CV trenches for FISH staining were imaged using the 20x objective. Z-stack images consisted of 20–35 stacks at 0.6 μm steps.

ImageJ (FIJI) was used for all immuno-stained tissues. Taste bud area profile ROIs were determined by KRT8 staining and outlined using the lasso tool. Taste cell counting was accomplished by moving through a z-stack, one slice at a time, counting partial and whole cells. Partial and whole cell profiles were counted as one cell. This method ensured cells were not counted more than once as taste cells are elongated and move through many z-slices. Images containing spotty staining were modified using the median filter in ImageJ, making the cells easily discernable. To measure taste cell and gustatory nerve fiber volume for each taste bud section, the z-stack fluorescence was adjusted to a median brightness with a 1.0 pixel radius and a threshold was set to encompass all positive signal. The same ROI used to determine bud area was used to measure volume. Using a macro (https://visikol.com/blog/2018/11/29/blog-post-loading-and-measurement-of-volumes-in-3d-confocal-image-stacks-with-imagej/), the thresholded z-stack was converted from voxels to μm^3^. Counting CALHM1 and Bassoon puncta was accomplished moving through each Z-slice in a Z-stack using the magic wand tracing tool in ImageJ. CALHM1 and Bassoon puncta greater than 0.5 μm and small puncta clusters were counted as 1 individual puncta. Most puncta spanned across more than one section in the z-stack, as our step-size was 0.6 μm. Puncta per bud was determined by counting all of the puncta within the taste bud in each 18 μm Z-stack. Contacted vs. uncontacted puncta were evaluated in each Z-slice in the Z-stack by their proximity to gustatory neurons using P2X2 staining. CALHM1 and Bassoon puncta that were overlapping with or directly touching P2X2 staining were categorized as “contacted puncta” and all others were characterized as “uncontacted”.

ImageJ (FIJI) was also used for in situ hybridization RNAscope images. Taste bud area profiles were determined by DAPI staining and outlined using the lasso tool. Since puncta were numerous and spanned across multiple sections of the z-stack, we utilized the ImageJ macro language to convert puncta to 3D objects. In brief, the despeckle function was applied to minimize background staining. A threshold using the Otsu dark algorithm was applied to select positive staining. Then, the 3D objects counter was used to connect thresholded puncta across z-stack sections. Puncta, in the form of 3D objects, were then quantified using the 3D ROI manager.

### Anesthesia and preparation of the mouse for intravital imaging

Mice were given an initial intraperitoneal (IP) injection of 10 mg/kg ketamine and 0.1 mg/kg xylazine (Covetrus). An IP injection was also given prior to positioning the mouse in the imaging apparatus to maintain fluid levels during imaging. The mouse was placed on their back and the tongue was inserted in the opening of a tongue holder using blunt forceps, as in [43]. Initially, a 10 × Olympus lens was used to spatially recognize that the same taste bud was being imaged. Once the correct bud was identified, a 40x water immersion lens was used to image the gustatory fibers. After each imaging session, the mouse was given an IP injection of Atipamezole (Covetrus) (1 mg/kg) to reverse the effects of the sedative.

### Two-photon imaging

Two-photon excitation microscopy imaging was used to obtain 3D images of a live mouse tongue, using a Bruker system with Prairie View software. A laser strength of approximately 400 mV and a wavelength of 1100 nm was used to visualize the tdTomato-labeled nerve fibers innervating the tongue. The same PMT and imaging strength were used each day to ensure no variation due to the setting changes. Images were collected at 1024 × 1024 pixels, resonance galvo, with resonance averaging at 2 frames. This resonance setting was used because it takes a faster image which is critical due to our time constraints of the sedative. Z stacks were collected at 0.5 μm intervals covering approximately a volume of 100 μm, cubed. Typically, most of the taste bud is imaged within the first 50 μm of the Z-axis, but additional images were collected toward the base of the bud to help orient the 3D volume.

### Three-dimensional image analysis

ImageJ was utilized for data analysis of 2-photon images to obtain a volume measurement of the fibers in pixels, cubed. First, a consistent threshold setting was used in ImageJ to minimize background fluorescence/autofluorescence. Then, an ROI was placed around the taste bud region to include only the region inside of the taste papillae. This region of interest included the bottom portion of the bud that comprises the dense papilla core, excluding the gustatory fibers beyond the basal lamina. A volume calculator in ImageJ was utilized to calculate the volume of fibers innervating each taste bud from each section of the z-stack.

## Supporting information

S1 Fig Bassoon and CALHM1 puncta colocalize with type III and type II cells, respectively. A) Immuno-labeling of KRT8 (blue), P2X2 (red), Car4 (yellow) and Bassoon (green). Bassoon staining is only seen at the junction of Car4 cells and P2X2 neurons. Bassoon staining is only seen at the junction of Car4 (type III) cells and P2X2 neurons. B) Immuno-labeling of Trpm5 (Magenta), P2X2 (red), and CALHM1 (green). CALHM1 staining is only seen at the junction of Trpm5 (type II) cells and P2X2 neurons. Scale: 10μm.
(PDF)

S2 Fig P2X2 immunostaining colocalizes with genetically driven Phox2b-Ai9 in regenerating gustatory neurons. Sectioned fungiform papilla from a Phox2b-Ai9 mouse 8 days following nerve transection. A) Immuno-labeling of KRT8 (blue), P2X2 (magenta), and Bassoon (green) and innate tdTomato fluorescent signal from Phox2b-Ai9 (red). B) Immuno-labeling of KRT8 (blue), P2X2 (magenta), and CALHM1 (green) and innate tdTomato fluorescent signal from Phox2b-Ai9 (red). Scale: 10μm.
(PDF)

S1 File Data files used for the production of the main figures. (ZIP)

## References

[1] Kashio M, Wei-Qi G, Ohsaki Y, Kido MA, Taruno A. CALHM1/CALHM3 channel is intrinsically sorted to the basolateral membrane of epithelial cells including taste cells. Sci Rep. 2019;9(1):2681. doi: 10.1038/s41598-019-39593-5 30804437 PMC6390109

[2] Romanov RA, Lasher RS, High B, Savidge LE, Lawson A, Rogachevskaja OA, et al. Chemical synapses without synaptic vesicles: purinergic neurotransmission through a CALHM1 channel-mitochondrial signaling complex. Sci Signal. 2018;11(529):eaao1815. doi: 10.1126/scisignal.aao1815 29739879 PMC5966022

[3] Ma Z, Taruno A, Ohmoto M, Jyotaki M, Lim JC, Miyazaki H, et al. CALHM3 is essential for rapid ion channel-mediated purinergic neurotransmission of GPCR-mediated tastes. Neuron. 2018;98(3):547-561.e10. doi: 10.1016/j.neuron.2018.03.043 29681531 PMC5934295

[4] Taruno A, Vingtdeux V, Ohmoto M, Ma Z, Dvoryanchikov G, Li A, et al. CALHM1 ion channel mediates purinergic neurotransmission of sweet, bitter and umami tastes. Nature. 2013;495(7440):223–6. doi: 10.1038/nature11906 23467090 PMC3600154

[5] Yang R, Crowley HH, Rock ME, Kinnamon JC. Taste cells with synapses in rat circumvallate papillae display SNAP-25-like immunoreactivity. J Comp Neurol. 2000;424(2):205–15. doi: 10.1002/1096-9861(20000821)424:2<205::aid-cne2>3.0.co;2-f 10906698

[6] Huang YA, Maruyama Y, Stimac R, Roper SD. Presynaptic (Type III) cells in mouse taste buds sense sour (acid) taste. J Physiol. 2008;586(12):2903–12. doi: 10.1113/jphysiol.2008.151233 18420705 PMC2517205

[7] Liman ER, Kinnamon SC. Sour taste: receptors, cells and circuits. Curr Opin Physiol. 2021;20:8–15. doi: 10.1016/j.cophys.2020.12.006 33709046 PMC7943026

[8] Ikuta R, Hamada S. The presynaptic active zone protein Bassoon as a marker for synapses between Type III cells and afferent nerve fibers in taste buds. Chem Senses. 2022;47:bjac016. doi: 10.1093/chemse/bjac016 35762653

[9] Asano-Miyoshi M, Hamamichi R, Emori Y. Synaptophysin as a probable component of neurotransmission occurring in taste receptor cells. J Mol Histol. 2009;40(1):59–70. doi: 10.1007/s10735-009-9214-5 19253017

[10] Kohno R, Toyono T, Seta Y, Kataoka S, Yamaguchi K, Toyoshima K. Expression of synaptotagmin 1 in the taste buds of rat gustatory papillae. Arch Histol Cytol. 2005;68(4):235–41. doi: 10.1679/aohc.68.235 16477143

[11] Kotani T, Toyono T, Seta Y, Kitou A, Kataoka S, Toyoshima K. Expression of synaptogyrin-1 in T1R2-expressing type II taste cells and type III taste cells of rat circumvallate taste buds. Cell Tissue Res. 2013;353(3):391–8. doi: 10.1007/s00441-013-1629-3 23636420

[12] Yang R, Ma H, Thomas SM, Kinnamon JC. Immunocytochemical analysis of syntaxin-1 in rat circumvallate taste buds. J Comp Neurol. 2007;502(6):883–93. doi: 10.1002/cne.21317 17447252

[13] Yang R, Dzowo YK, Wilson CE, Russell RL, Kidd GJ, Salcedo E, et al. Three-dimensional reconstructions of mouse circumvallate taste buds using serial blockface scanning electron microscopy: I. Cell types and the apical region of the taste bud. J Comp Neurol. 2020;528(5):756–71. doi: 10.1002/cne.24779 31587284 PMC7041425

[14] Finger TE, Danilova V, Barrows J, Bartel DL, Vigers AJ, Stone L, et al. ATP signaling is crucial for communication from taste buds to gustatory nerves. Science. 2005;310(5753):1495–9. doi: 10.1126/science.1118435 16322458

[15] Huang YA, Stone LM, Pereira E, Yang R, Kinnamon JC, Dvoryanchikov G, et al. Knocking out P2X receptors reduces transmitter secretion in taste buds. J Neurosci. 2011;31(38):13654–61. doi: 10.1523/JNEUROSCI.3356-11.2011 21940456 PMC3188419

[16] Vandenbeuch A, Larson ED, Anderson CB, Smith SA, Ford AP, Finger TE, et al. Postsynaptic P2X3-containing receptors in gustatory nerve fibres mediate responses to all taste qualities in mice. J Physiol. 2015;593(5):1113–25. doi: 10.1113/jphysiol.2014.281014 25524179 PMC4358674

[17] Bo X, Alavi A, Xiang Z, Oglesby I, Ford A, Burnstock G. Localization of ATP-gated P2X2 and P2X3 receptor immunoreactive nerves in rat taste buds. Neuroreport. 1999;10(5):1107–11. doi: 10.1097/00001756-199904060-00037 10321492

[18] Eddy MC, Eschle BK, Barrows J, Hallock RM, Finger TE, Delay ER. Double P2X2/P2X3 purinergic receptor knockout mice do not taste NaCl or the artificial sweetener SC45647. Chem Senses. 2009;34(9):789–97. doi: 10.1093/chemse/bjp068 19833661 PMC2762055

[19] Larson ED, Vandenbeuch A, Voigt A, Meyerhof W, Kinnamon SC, Finger TE. The role of 5-HT3 receptors in signaling from taste buds to nerves. J Neurosci. 2015;35(48):15984–95. doi: 10.1523/JNEUROSCI.1868-15.2015 26631478 PMC4666921

[20] Flammer LJ, Ellis H, Rivers N, Caronia L, Ghidewon MY, Christensen CM, et al. Topical application of a P2X2/P2X3 purine receptor inhibitor suppresses the bitter taste of medicines and other taste qualities. British J Pharmacol. 2024;181(17):3282–99. doi: 10.1111/bph.16411 38745397

[21] Wilson CE, Lasher RS, Yang R, Dzowo Y, Kinnamon JC, Finger TE. Taste bud connectome: implications for taste information processing. J Neurosci. 2021;42(5):804–16. doi: 10.1523/jneurosci.0838-21.2021 34876471 PMC8808728

[22] Beidler LM, Smallman RL. Renewal of cells within taste buds. J Cell Biol. 1965;27(2):263–72. doi: 10.1083/jcb.27.2.263 5884625 PMC2106718

[23] Südhof TC. Towards an understanding of synapse formation. Neuron. 2018;100(2):276–93. doi: 10.1016/j.neuron.2018.09.040 30359597 PMC6226307

[24] Südhof TC. The cell biology of synapse formation. J Cell Biol. 2021;220(7):e202103052. doi: 10.1083/jcb.202103052 34086051 PMC8186004

[25] Ishida Y, Ugawa S, Ueda T, Yamada T, Shibata Y, Hondoh A, et al. P2X(2)- and P2X(3)-positive fibers in fungiform papillae originate from the chorda tympani but not the trigeminal nerve in rats and mice. J Comp Neurol. 2009;514(2):131–44. doi: 10.1002/cne.22000 19266560

[26] Knapp L, Lawton A, Oakley B, Wong L, Zhang C. Keratins as markers of differentiated taste cells of the rat. Differentiation. 1995;58(5):341–9. doi: 10.1046/j.1432-0436.1995.5850341.x 7542613

[27] Mbiene J-P, Roberts JD. Distribution of keratin 8-containing cell clusters in mouse embryonic tongue: evidence for a prepattern for taste bud development. J Comp Neurol. 2003;457(2):111–22. doi: 10.1002/cne.10551 12541313

[28] Ogata T, Ohtubo Y. Quantitative analysis of taste bud cell numbers in the circumvallate and foliate taste buds of mice. Chem Senses. 2020;45(4):261–73. doi: 10.1093/chemse/bjaa017 32157267

[29] Lin X, Lu C, Ohmoto M, Choma K, Margolskee RF, Matsumoto I, et al. R-spondin substitutes for neuronal input for taste cell regeneration in adult mice. Proc Natl Acad Sci U S A. 2021;118(2):e2001833118. doi: 10.1073/pnas.2001833118 33443181 PMC7812810

[30] Hendricks SJ, Sollars SI, Hill DL. Injury-induced functional plasticity in the peripheral gustatory system. J Neurosci. 2002;22(19):8607–13. doi: 10.1523/JNEUROSCI.22-19-08607.2002 12351734 PMC6757794

[31] Guagliardo NA, Hill DL. Fungiform taste bud degeneration in C57BL/6J mice following chorda-lingual nerve transection. J Comp Neurol. 2007;504(2):206–16. doi: 10.1002/cne.21436 17626272 PMC2811721

[32] Li Y-K, Yang J-M, Huang Y-B, Ren D-D, Chi F-L. Shrinkage of ipsilateral taste buds and hyperplasia of contralateral taste buds following chorda tympani nerve transection. Neural Regen Res. 2015;10(6):989–95. doi: 10.4103/1673-5374.158366 26199619 PMC4498364

[33] St John SJ, Garcea M, Spector AC. The time course of taste bud regeneration after glossopharyngeal or greater superficial petrosal nerve transection in rats. Chem Senses. 2003;28(1):33–43. doi: 10.1093/chemse/28.1.33 12502522

[34] Guth L. The effects of glossopharyngeal nerve transection on the circumvallate papilla of the rat. Anat Rec. 1957;128(4):715–31. doi: 10.1002/ar.1091280406 13478886

[35] Perea-Martinez I, Nagai T, Chaudhari N. Functional cell types in taste buds have distinct longevities. PLoS One. 2013;8(1):e53399. doi: 10.1371/journal.pone.0053399 23320081 PMC3540047

[36] Cheal M, Oakley B. Regeneration of fungiform taste buds: temporal and spatial characteristics. J Comp Neurol. 1977;172(4):609–26. doi: 10.1002/cne.901720405 838892

[37] Cheal M, Dickey WP, Jones LB, Oakley B. Taste fiber responses during reinnervation of fungiform papillae. J Comp Neurol. 1977;172(4):627–46. doi: 10.1002/cne.901720406 838893

[38] Meng L, Huang T, Sun C, Hill DL, Krimm R. BDNF is required for taste axon regeneration following unilateral chorda tympani nerve section. Exp Neurol. 2017;293:27–42. doi: 10.1016/j.expneurol.2017.03.016 28347764 PMC5625336

[39] Dong G, Kogan S, Venugopal N, Chang E, He L, Faal F, et al. Interleukin (IL)-1 receptor signaling is required for complete taste bud regeneration and the recovery of neural taste responses following axotomy. J Neurosci. 2023;43(19):3439–55. doi: 10.1523/JNEUROSCI.1355-22.2023 37015809 PMC10184746

[40] Walters BN, Whiddon ZD, McGee AW, Krimm RF. Longitudinal imaging of the taste bud in vivo with two-photon laser scanning microscopy. PLoS One. 2024;19(12):e0309366. doi: 10.1371/journal.pone.0309366 39671398 PMC11642993

[41] Whiddon ZD, Marshall JB, Alston DC, McGee AW, Krimm RF. Rapid structural remodeling of peripheral taste neurons is independent of taste cell turnover. PLoS Biol. 2023;21(8):e3002271. doi: 10.1371/journal.pbio.3002271 37651406 PMC10499261

[42] Huang T, Ohman LC, Clements AV, Whiddon ZD, Krimm RF. Variable branching characteristics of peripheral taste neurons indicates differential convergence. J Neurosci. 2021;41(22):4850–66. doi: 10.1523/jneurosci.1935-20.2021 33875572 PMC8260161

[43] Wood RM, Vasquez EL, Goyins KA, Gutierrez Kuri E, Connelly K, Humayun S, et al. Cyclophosphamide induces the loss of taste bud innervation in mice. Chemical Senses. 2024;49. doi: 10.1093/chemse/bjae010 PMC10929424 38421250

[44] Takeda N, Jain R, Li D, Li L, Lu MM, Epstein JA. Lgr5 identifies progenitor cells capable of taste bud regeneration after injury. PLoS One. 2013;8(6):e66314. doi: 10.1371/journal.pone.0066314 23824276 PMC3688887

[45] Yee KK, Li Y, Redding KM, Iwatsuki K, Margolskee RF, Jiang P. Lgr5-EGFP marks taste bud stem/progenitor cells in posterior tongue. Stem Cells. 2013;31(5):992–1000. doi: 10.1002/stem.1338 23377989 PMC3637415

[46] Ren W, Lewandowski BC, Watson J, Aihara E, Iwatsuki K, Bachmanov AA, et al. Single Lgr5- or Lgr6-expressing taste stem/progenitor cells generate taste bud cells ex vivo. Proc Natl Acad Sci U S A. 2014;111(46):16401–6. doi: 10.1073/pnas.1409064111 25368147 PMC4246268

[47] Kim HY, Um JW, Ko J. Proper synaptic adhesion signaling in the control of neural circuit architecture and brain function. Prog Neurobiol. 2021;200:101983. doi: 10.1016/j.pneurobio.2020.101983 33422662

[48] Dvoryanchikov G, Huang YA, Barro-Soria R, Chaudhari N, Roper SD. GABA, its receptors, and GABAergic inhibition in mouse taste buds. J Neurosci. 2011;31(15):5782–91. doi: 10.1523/JNEUROSCI.5559-10.2011 21490220 PMC3320853

[49] Dvoryanchikov G, Hernandez D, Roebber JK, Hill DL, Roper SD, Chaudhari N. Transcriptomes and neurotransmitter profiles of classes of gustatory and somatosensory neurons in the geniculate ganglion. Nat Commun. 2017;8(1):760. doi: 10.1038/s41467-017-01095-1 28970527 PMC5624912

[50] Panzanelli P, Früh S, Fritschy J-M. Differential role of GABAA receptors and neuroligin 2 for perisomatic GABAergic synapse formation in the hippocampus. Brain Struct Funct. 2017;222(9):4149–61. doi: 10.1007/s00429-017-1462-7 28643105

[51] Lee H, Macpherson LJ, Parada CA, Zuker CS, Ryba NJP. Rewiring the taste system. Nature. 2017;548(7667):330–3. doi: 10.1038/nature23299 28792937 PMC5805144

[52] Hirose F, Takai S, Takahashi I, Shigemura N. Expression of protocadherin-20 in mouse taste buds. Sci Rep. 2020;10(1):2051. doi: 10.1038/s41598-020-58991-8 32029864 PMC7005180

[53] Attardo A, Fitzgerald JE, Schnitzer MJ. Impermanence of dendritic spines in live adult CA1 hippocampus. Nature. 2015;523(7562):592–6. doi: 10.1038/nature14467 26098371 PMC4648621

[54] Ohman LC, Hanbali L, Krimm RF. Taste arbor structural variability analyzed across taste regions. J Comp Neurol. 2023;531(7):743–58. doi: 10.1002/cne.25459 36740741 PMC10082444

[55] Cai Q, Pan P-Y, Sheng Z-H. Syntabulin–Kinesin-1 family member 5B-mediated axonal transport contributes to activity-dependent presynaptic assembly. J Neurosci. 2007;27(27):7284–96. doi: 10.1523/jneurosci.0731-07.2007 17611281 PMC6794594

[56] Talagas M. Anatomical contacts between sensory neurons and epidermal cells: an unrecognized anatomical network for neuro-immuno-cutaneous crosstalk. Br J Dermatol. 2023;188(2):176–85. doi: 10.1093/bjd/ljac066 36763869

[57] Kaelberer MM, Buchanan KL, Klein ME, Barth BB, Montoya MM, Shen X, et al. A gut-brain neural circuit for nutrient sensory transduction. Science. 2018;361(6408):eaat5236. doi: 10.1126/science.aat5236 30237325 PMC6417812

[58] Padalhin A, Abueva C, Park SY, Ryu HS, Lee H, Kim JI, et al. Recovery of sweet taste preference in adult rats following bilateral chorda tympani nerve transection. PeerJ. 2022;10:e14455. doi: 10.7717/peerj.14455 36452076 PMC9703994

[59] McCluskey LP. Up-regulation of activated macrophages in response to degeneration in the taste system: effects of dietary sodium restriction. J Comp Neurol. 2004;479(1):43–55. doi: 10.1002/cne.20307 15389612

