# Supplementary figures and images for: Taste cells depend on axon proximity to generate presynaptic sites
Source: PLoS One. 2025 Jun 3;20(6):e0325312. doi: 10.1371/journal.pone.0325312 (PMC12132963; doi:10.1371/journal.pone.0325312)

A

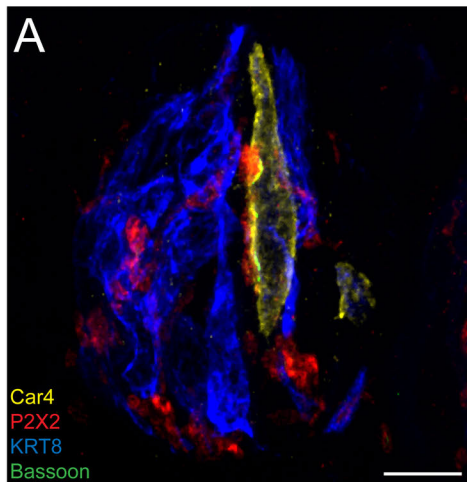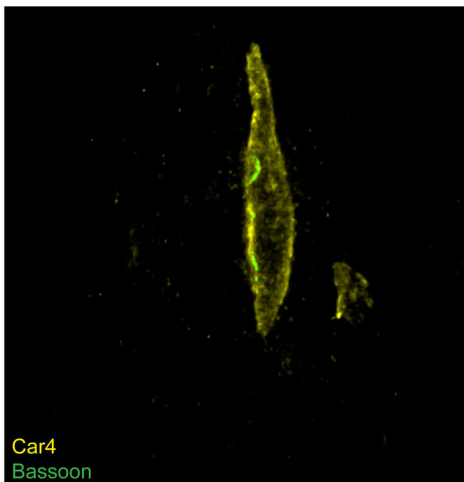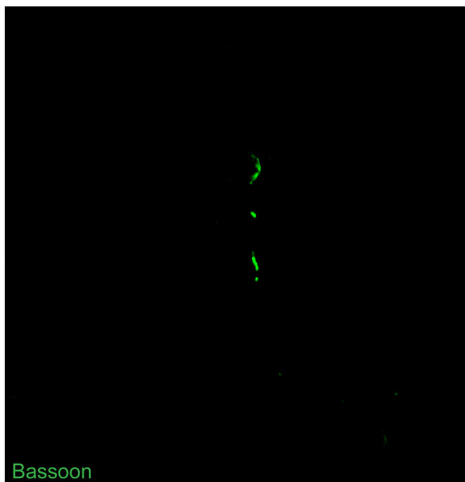

B

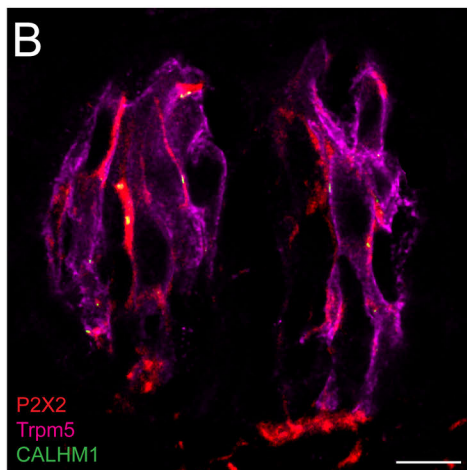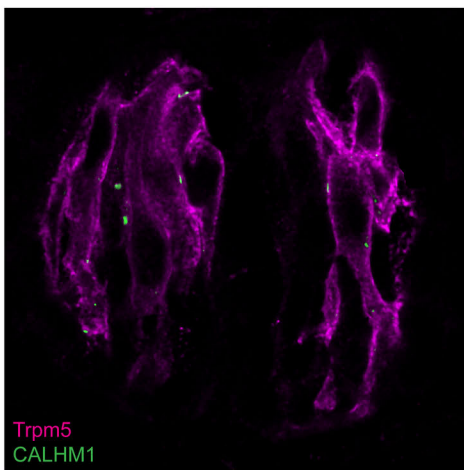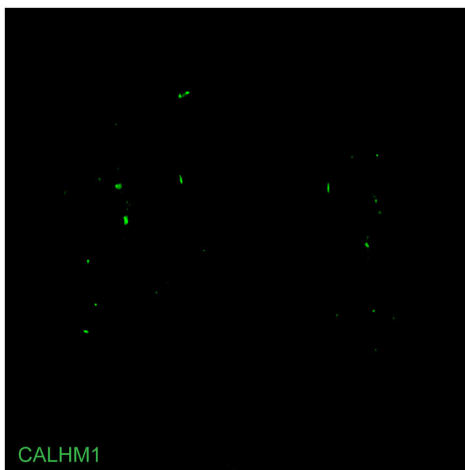

Supplement: S1 Fig — A) Immuno-labeling of KRT8 (blue), P2X2 (red), Car4 (yellow) and Bassoon (green). Bassoon staining is only seen at the junction of Car4 cells and P2X2 neurons. Bassoon staining is only seen at the junction of Car4 (type III) cells and P2X2 neurons. B) Immuno-labeling of Trpm5 (Magenta), P2X2 (red), and CALHM1 (green). CALHM1 staining is only seen at the junction of Trpm5 (type II) cells and P2X2 neurons. Scale: 10μm. (PDF) [file pone.0325312.s001.pdf]

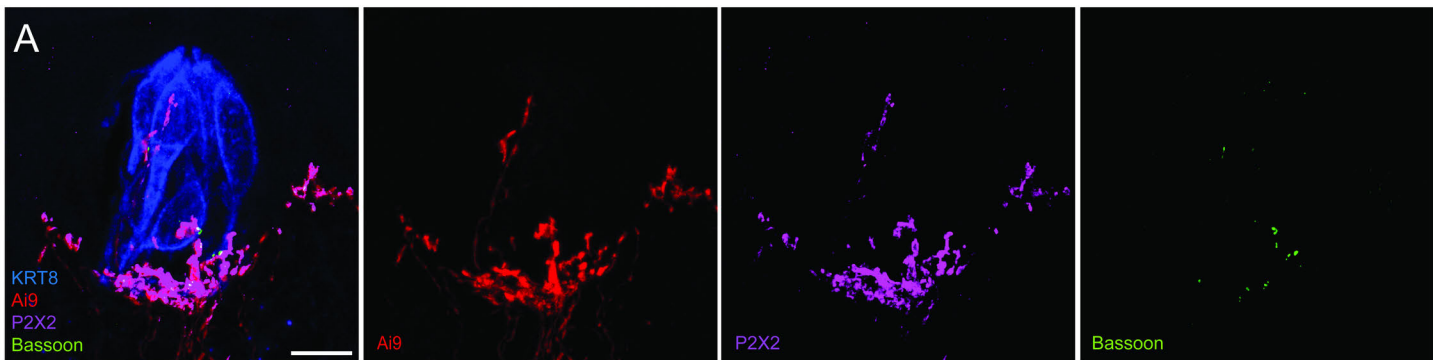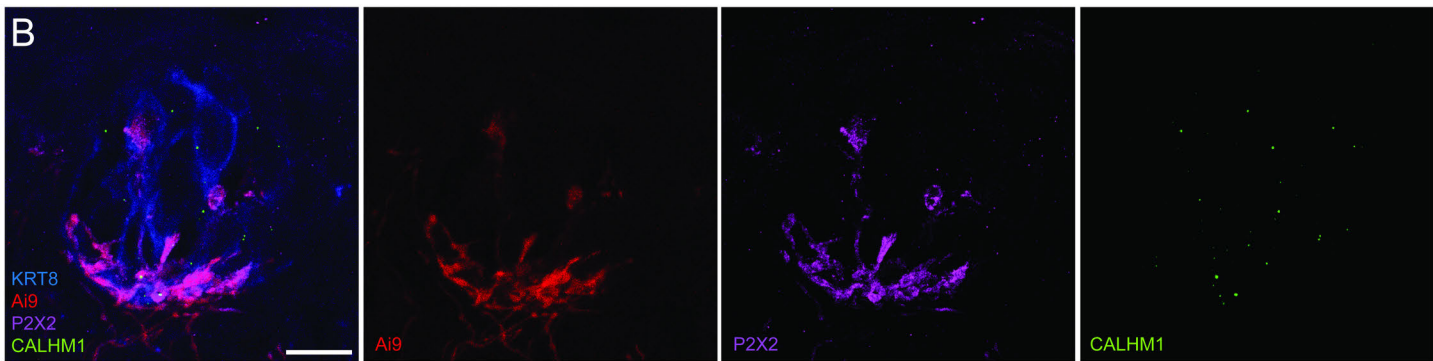

Supplement: S2 Fig — Sectioned fungiform papilla from a Phox2b-Ai9 mouse 8 days following nerve transection. A) Immuno-labeling of KRT8 (blue), P2X2 (magenta), and Bassoon (green) and innate tdTomato fluorescent signal from Phox2b-Ai9 (red). B) Immuno-labeling of KRT8 (blue), P2X2 (magenta), and CALHM1 (green) and innate tdTomato fluorescent signal from Phox2b-Ai9 (red). Scale: 10μm. (PDF) [file pone.0325312.s002.pdf]
